# Supplementary material for: Mental Health Problems among Young People—A Scoping Review of Help-Seeking
Source: Int J Environ Res Public Health. 2022 Jan 27;19(3):1430. doi: 10.3390/ijerph19031430 (PMC8835517; doi:10.3390/ijerph19031430)
Supplement: Supplementary file 1 [file ijerph-19-01430-s001.zip › Table S1. Full search strategy .pdf]

Table S1. Full search strategy.

	Cinahl	MedLine/PubMed	PsycINFO
**Date**	2020-08-13	2020-07-08	2020-09-03
Mental health (MESH) + adolescence/adolescent (MESH) + help-seeking behavior (MESH)	88	26	
Behavioral Symptoms [Mesh]) AND Help-Seeking Behavior [Mesh]) AND Adolescence/adolescent[Mesh]	389	99	
Psychological distress (MESH) + help-seeking behavior AND Adolescence/adolescent(MESH)	1		
Mental health (MESH) + young adult (MESH) help-seeking behavior (MESH)	26	31	
Behavioral Symptoms[Mesh]) AND Help-Seeking Behavior[Mesh]) AND Young Adult [Mesh]	177	103	
Psychological distress (MESH) + help-seeking behavior AND Young adult (MESH)	1		
Psychological distress (MESH) + help-seeking behavior (MESH)		3	
Depression [Mesh]) AND "Help-Seeking Behavior"[Mesh]) AND "Adolescent"[Mesh]		35	
Depression [Mesh] AND ”Help-Seeking Behavior"[Mesh] AND "Young Adult”[Mesh]		47	
Anxiety [Mesh]) AND "Help-Seeking Behavior"[Mesh]) AND "Adolescent"[Mesh]		14	
Anxiety [Mesh] AND "Help-Seeking Behavior" AND “Young Adult"[Mesh]		17	
Suicidal Ideation"[Mesh]) AND "Help-Seeking Behavior"[Mesh]) AND "Adolescent"[Mesh]		24	
Suicidal Ideation"[Mesh]) AND "Help-Seeking Behavior"[Mesh]) AND "Young Adult"[Mesh]		19	
Self-Injurious Behavior"[Mesh]) AND "Help-Seeking Behavior"[Mesh]) AND "Adolescent"[Mesh]		50	
Self-Injurious Behavior"[Mesh]) AND "Help-Seeking Behavior"[Mesh]) AND "Young Adult"[Mesh]		45	
“Mental health” AND “help-seeking” AND adolescen* fritextsökning		819	
“Mental health” AND “help-seeking” AND young fritextsökning		856	

Mental health (MESH) + help-seeking behavior (MESH) + adolescent attitudes [MESH]			13
Mental health (MESH) + help-seeking behavior (MESH) + adolescent behavior (MESH)			3
Mental health (MESH) +help-seeking behavior (MESH)+adolescent characteristics (MESH)			1
Mental health (MESH) + help-seeking behavior (MESH) + adolescent health (MESH)			2
Mental health (MESH) + help-seeking behavior (MESH) + emerging adulthood (MESH)			3
Depression, emotion (MESH) + help-seeking behavior (MESH) adolescent attitudes (MESH)			1
Depression, emotion (MESH) + help-seeking behavior (MESH) adolescent behavior (MESH)			1
Depression, emotion (MESH) + help-seeking behavior (MESH) adolescent characteristics (MESH)			0
Depression, emotion (MESH) +help-seeking behavior (MESH)+adolescent health (MESH)			0
Depression, emotion (MESH) +help-seeking behavior(MESH)+emerging adulthood (MESH)			1
Anxiety (MESH) + help-seeking behavior (MESH) + adolescent attitudes (MESH)			1
Anxiety (MESH) + help-seeking behavior (MESH) + adolescent behavior (MESH)			0
Anxiety (MESH) + help-seeking behavior (MESH) + adolescent characteristics (MESH)			1
Anxiety (MESH) + help-seeking behavior (MESH) + adolescent health (MESH)			1
Anxiety (MESH) + help-seeking behavior (MESH) + emerging adulthood (MESH)			0
Self-destructive behavior (MESH) + help-seeking behavior (MESH) + adolescent attitudes (MESH)			3
Self-destructive behavior (MESH) + help-seeking behavior (MESH) + adolescent behavior (MESH)			1
Self-destructive behavior (MESH) + help-seeking behavior (MESH) + adolescent characteristics(MESH)			2
Self-destructive behavior (MESH) + help-seeking behavior (MESH) + adolescent health (MESH)			0
Self-destructive behavior (MESH) + help-seeking behavior (MESH) + emerging adulthood (MESH)			1
**Combined search**<br>**Limits:** 2010 -2020, English	682	2188	35
**Total**	2905		
**Total minus duplicates**	1540		
