# Supplementary material for: Mental Health Problems among Young People—A Scoping Review of Help-Seeking
Source: Int J Environ Res Public Health. 2022 Jan 27;19(3):1430. doi: 10.3390/ijerph19031430 (PMC8835517; doi:10.3390/ijerph19031430)
Supplement: Supplementary file 1 [file ijerph-19-01430-s001.zip › Table S2. Table of included studies N=12.pdf]

Table S2: Characteristics of included studies.

**Author**	**Title**	**Journal**	**Year**	**Country**	**Aim**	**Design**	**Sample (study population and age)**	**Main results**
Anyon, Y. et al.	Help-Seeking in the School Context: Understanding Chinese American Adolescents' Underutilization of School Health Services	Journal of School Health 2013 Vol. 83 Issue 8 Pages 562-572	2013	USA	To identify and investigate social and organizational factors unique to Chinese American students' help-seeking process in a school context	Sequential mixed methods; cross-sectional survey followed by a grounded theory approach with focus groups and individual interviews. Recruited in schools.	Stated according to high school grades 9-12, equivalent of ages 14-18. Survey N= 1744 Qualitative part N=51	Contextual influences shaped students' help-seeking trajectories in schools more than attitudinal and behavioural barriers. Chinese American students' constrained self-identification hinder help-seeking in schools.
Cairns, A. et al.	Exploring functional concerns in help-seeking youth: a qualitative study	Early Interv Psychiatry 2015 Vol. 9 Issue 3 Pages 228-33	2015	Australia	To explore functional concerns of help-seeking young people	Qualitative, semi-structured interviews, content analysis. Recruited at youth mental health clinic.	Age 14-25 N=10	Identified four themes for seeking help; relationships, emotional problems, risk-taking behaviour and employment concerns. Themes differed somewhat according to age.
Clark, L. H. et al.	Capturing the Attitudes of Adolescent Males' Towards Computerised Mental Health Help-Seeking	Australian Psychologist 2018 Vol. 53 Issue 5 Pages 416-426	2018	Australia	To explore the attitudes of young males towards computerized mental health support and treatment	Qualitative, semi-structured interviews and focus groups, content analysis.	Age 12-18 N=29	Barriers for computerized mental health interventions were unfamiliarity, control over decision-making, effort involved and confidentiality. Barriers were found to be much the same as

						Recruited at child and adolescent mental health services, schools and community website.		barriers for accessing other kinds of support, however, it was still suggested that computerized mental health support was more accessible.
Corry, D. A., Leavey, G.	Adolescent trust and primary care: Help-seeking for emotional and psychological difficulties	J Adolesc 2017 Vol. 54 Pages 1-8	2017	N Ireland	To explore adolescents' attitudes to consulting their GP for psychological problems.	Qualitative, focus groups, thematic analysis. Recruited in schools.	Age 13-16<br><br>N=54	A general reluctance to engage with the GP was noted, primarily due to a pervasive lack of trust. Girls expressed being more anxious about seeking help from GPs whereas older boys were particularly concerned about confidentiality.
Lindsey, M. A. et al.	Understanding the Behavioral Determinants of Mental Health Service Use by Urban, Under-Resourced Black Youth: Adolescent and Caregiver Perspectives	J Child Fam Stud 2013 Vol. 22 Issue 1 Pages 107-121	2013	USA	To explore mental health help-seeking behaviours and perceptions of mental health services in schools among urban, under-resourced Black youth, applying the Unified Theory of Behaviour as theoretical construct.	Qualitative, focus groups. Recruited in schools by a school mental health clinician.	Age 11-14 N=16 (+11 caregivers)	Negative expectancies and social norms regarding mental health treatment were seen to influence service use negatively.
Lynch, L. et al.	Young Men, Help-Seeking, and Mental Health Services: Exploring Barriers and Solutions	Am J Mens Health 2018 Vol. 12 Issue 1 Pages 138-149	2018	Ireland	To explore barriers to, and solutions for, professional help-seeking for mental health problems amongst young men.	Qualitative, individual interviews and focus groups, thematic analysis. Recruited at local youth service.	Age 18-24<br><br>N=17	Key themes of barriers touched on both personal and environmental factors. Important findings were that young men fear psychiatric medication and homophobic responses from professionals, and highlights the

								need for implementing mental health education.
Martinez-Harnaez, A. et al.	Non-professional-help-seeking among young people with depression: a qualitative study	BMC Psychiatry 2014 Vol. 14 Pages 124	2014	Spain	To explore reasons for non-professional help-seeking and obtain recommendations for improving access to mental health care services.	Qualitative, individual interviews and focus groups<br>Recruited through previous participation in a longitudinal study.	Age 17-21<br><br>N=105	Reasons for not seeking help were strongly conditioned by gender. A three-phase model was proposed of the help-seeking process; normalization, problematization and evaluation of consequences.
Rice, S.M.et al.	Young men's access to community-based mental health care: qualitative analysis of barriers and facilitators	J Ment Health 2018 Vol. 27 Issue 1 Pages 59-65	2018	Australia	To identify barriers and facilitators to mental health care as identified by young men with a recent history of help-seeking.	Qualitative, individual interviews and focus groups, thematic analysis.<br>Recruited at youth-specific early intervention centres.	Age 12-25<br><br>N=25<br>(25 young men + professional supporters)	Identified barriers to seeking help were; male role expectations, unfamiliarity with talk therapy, difficulties navigating the system, intake processes. Facilitators were; positive initial contact, effective cross-sector partnerships, availability of male supporters and use of targeted messaging.
Salaheddin, K. and Mason, B.	Identifying barriers to mental health help-seeking among young adults in the UK: a cross-sectional survey	Br J Gen Pract 2016 Vol. 66 Issue 651 Pages e686-92	2016	UK	To explore barriers in accessing mental health support among young people.	Quantitative, cross-sectional survey + open ended question with thematic analysis.<br>Community sample.	Age 18-25<br><br>N=203	More than 1/3 of participants did not seek help despite having emotional or mental health problems. Major barriers to help were; stigmatising beliefs, difficulties identifying or articulating concerns, a preference for self-reliance and difficulties accessing help.

Spence, R. et al.	Help-seeking in emerging adults with and without a history of mental health referral: a qualitative study	BMC Res Notes 2016 Vol. 9 Issue 1 Pages 415	2016	UK	To explore help-seeking strategies in young adults with or without a history of referrals, compare differences between groups and explore barriers to care.	Qualitative, semi-structured interviews, thematic analysis. Recruited from a longitudinal study.	Age 20-22<br><br>N=29	Young people used a combination of approach and avoidant techniques for dealing with mental health problems. Those with a history of referral to support services suffered more from stigma and were more likely to rely on avoidant or suppression techniques which was interpreted as having consequences for choice of help-seeking strategies.
Stafford, A.M. et al.	Getting a Grip on My Depression: How Latina Adolescents Experience, Self-Manage, and Seek Treatment for Depressive Symptoms	Qualitative Health Research 2019 Vol. 29 Issue 12 Pages 1725-1738	2019	USA	To develop a framework that describe experience, self-management and treatment-seeking for depressive symptoms by Latina adolescents	Qualitative, semi-structured interviews, grounded theory. Recruited from primary care and community settings.	Age 13-20<br><br>N=25	A five-phase psychosocial process was found to depict the process of experiencing, self-managing and seeking help.
Tang, M.O. et al.	College men's depression-related help-seeking: a gender analysis	J Ment Health 2014 Vol. 23 Issue 5 Pages 219-24	2014	Canada	To describe connections between masculinities and help-seeking of college men with depression.	Qualitative, semi-structured interviews, interpretive description. Recruited at university.	Age 19-25<br><br>N=21	Three themes were identified; denying weakness, limiting self-disclosure and mastering autonomy, redefining strength
